# Evolution Shapes the Gene Expression Response to Oxidative Stress

**DOI:** 10.3390/ijms20123040

**Published:** 2019-06-21

**Authors:** Rima Siauciunaite, Nicholas S. Foulkes, Viola Calabrò, Daniela Vallone

**Affiliations:** 1 Institute of Toxicology and Genetics, Karlsruhe Institute of Technology, Hermann-von-Helmholtz-Platz 1, 76344 Eggenstein-Leopoldshafen, Germany; rima.siauciunaite@kit.edu (R.S.); nicholas.foulkes@kit.edu (N.S.F.); 2 Department of Biology, Monte Sant’Angelo Campus, University of Naples Federico II, Via Cinthia 4, 80126 Naples, Italy; vcalabro@unina.it

**Keywords:** ROS, light, DNA damage, evolution, D-box, cavefish, *Spalax*

## Abstract

Reactive oxygen species (ROS) play a key role in cell physiology and function. ROS represents a potential source of damage for many macromolecules including DNA. It is thought that daily changes in oxidative stress levels were an important early factor driving evolution of the circadian clock which enables organisms to predict changes in ROS levels before they actually occur and thereby optimally coordinate survival strategies. It is clear that ROS, at relatively low levels, can serve as an important signaling molecule and also serves as a key regulator of gene expression. Therefore, the mechanisms that have evolved to survive or harness these effects of ROS are ancient evolutionary adaptations that are tightly interconnected with most aspects of cellular physiology. Our understanding of these mechanisms has been mainly based on studies using a relatively small group of genetic models. However, we know comparatively little about how these mechanisms are conserved or have adapted during evolution under different environmental conditions. In this review, we describe recent work that has revealed significant species-specific differences in the gene expression response to ROS by exploring diverse organisms. This evidence supports the notion that during evolution, rather than being highly conserved, there is inherent plasticity in the molecular mechanisms responding to oxidative stress.

## 1. Background

Since the origin of life on earth, oxidative stress has posed a major challenge for living systems. From the evolution of the first plants and photosynthesis to the development of aerobic oxidative respiration, living systems have faced the challenge of exposure to elevated oxygen levels and consequently Reactive Oxygen Species (ROS) (including peroxides (e.g., H_2_O_2_), superoxide (O_2_^•−^), hydroxyl radicals (^•^OH) and singlet oxygen (^1^O_2_) [1]). Oxidative stress accompanies exposure to environmental stressors such as hypoxia, UV radiation, as well as visible light and so frequently changes across the day-night cycle. In more recent evolutionary time, in relation to the impact of human activities on the environment, the toxic effects of many man-made compounds also induce oxidative stress. 

ROS levels are modulated by a balance between pro-oxidant and antioxidant elements. When increased levels of ROS are not countered by increases of antioxidant activity or reducing equivalents, a cell undergoes an oxidative stress state. Higher concentrations of ROS represent a potential source of damage for many macromolecules due to the induction of single- and double-stranded DNA breaks, oxidative decarboxylation of α-ketoacids such as pyruvate, and irreversible denaturation of proteins through oxidation and carbonylation of arginine, proline, lysine, and threonine residues [2]. Many cellular mechanisms have evolved to counteract these effects which are based on enzymatic as well as non-enzymatic processes. One of the best described is the role of glutathione in its reduced state (GSH). GSH serves as an antioxidant in plants, animals, fungi and also in some bacteria. It neutralizes ROS by directly donating a reduced equivalent (H^+^ + e^−^). Furthermore, GSH activates oxidative stress response cascades by promoting transcription and post-translational modifications of proteins that affect their functionality. Another conserved antioxidant mechanism is based on the rapid rerouting of carbohydrate flux from glycolysis to the pentose phosphate pathway (PPP) via inhibiting the activity of glycolytic enzymes such as GAPDH (glyceraldehyde-3-phosphate dehydrogenase). The PPP pathway then has the role of regulating cellular NADPH levels that serve as the fuel for antioxidant systems [3,4,5]. 

Important antioxidant enzymes used in nearly all cells exposed to oxygen are the superoxide dismutases (SODs) and the catalases, as well as peroxiredoxins and glutathione peroxidases. SODs are a group of metalloproteins catalyzing the dismutation of superoxide O_2_^•−^ radicals into two less damaging species, O_2_ and H_2_O_2_. There are three major families of superoxide dismutase depending on the metal cofactor used. The SOD Cu/Zn family which binds copper and zinc is mainly used in eukaryotes including humans; the SOD families which either bind iron and manganese or nickel are used by prokaryotic and protozoa. The physiological importance of SODs is illustrated by the severe pathologies observed in genetically engineered model organisms lacking these enzymes spanning from mouse and *Drosophila* to yeast [6,7]. Hydrogen peroxide is subsequently degraded by catalase activity, usually localized in peroxisomes. This highly active enzyme that catalyzes the decomposition of millions of H_2_O_2_ molecules to water and oxygen each second [8], also plays a central role in aging and degenerative disorders in humans [9]. Other enzymes involved in scavenging H_2_O_2_ outside of the peroxisomes are the Peroxiredoxins [10] and the Glutathione peroxidases [11] that detoxify a broad range of peroxides to the corresponding alcohols or water.

Together with strategies aimed at reducing the levels of ROS, a key adaptation for surviving the harmful effects is the evolution of DNA repair mechanisms such as base excision repair, (BER) [12,13], which targets ROS-induced covalent modifications of bases. Several regulatory systems, including cell cycle control and apoptosis, also protect organisms from the negative effects of ROS and are themselves activated by oxidative stress. Cell fate decisions involving cell cycle arrest and apoptosis represent key cellular responses to the damaging effects of ROS. Furthermore, it is increasingly clear that ROS, at relatively low levels, can reversibly oxidize redox-sensitive cysteine and methionine residues, acting as a second messenger via the targeted inactivation of enzymes bearing active site cysteines, for example, phosphotyrosine phosphatases [14]. 

The gene expression control mechanisms that enable organisms to survive elevated ROS levels or harness their effects are frequently ancient evolutionary adaptations that are tightly interconnected with most aspects of cellular physiology. These mechanisms have received significant attention in studies involving a small number of genetically accessible model organisms. However, comparatively little is known about how these mechanisms are conserved or have adapted during evolution under different environmental conditions. 

Even the simplest unicellular organisms possess mechanisms to counter the damaging effects of oxidative stress. Plants and animals frequently excrete hydrogen peroxide or superoxide-generating redox-cycling compounds as a strategy to inhibit microbial growth [15,16] and so bacteria frequently inhabit oxidizing environments. They protect themselves by activating regulons controlled by the OxyR, PerR and, SoxR transcription factors [16,17,18]. In *Escherichia coli*, the SoxR transcription factor induces the expression of the SoxS protein that in turn activates the transcription of several other genes including the antioxidant enzyme superoxide dismutase [19]. In *Rhodobacter*, the redox signal is detected by the membrane-bound sensor kinase, RegB via a redox-active cysteine located in its cytosolic domain that in turn regulates autophosphorylation. The active form of RegB is able to phosphorylate its regulatory partner RegA, capable of activating or repressing a variety of genes [20]. The conservation of both RegB and RegA homologs in a broad range of bacteria points to a central role for the RegB/RegA two component system in the transcriptional regulation of redox-regulated gene expression [21]. 

In the yeast *Saccharomyces cerevisiae*, peroxiredoxins are ubiquitous, thiol-containing antioxidant proteins that serve to reduce hydroperoxides. They also regulate hydrogen peroxide-mediated signal transduction via activation of transcription factors such as NF-κB [22,23]. Particularly relevant for the antioxidant function of the peroxiredoxins is a conserved protein called Sulphiredoxin. Sulphiredoxin can reduce cysteine-sulphinic acid of the antioxidant peroxiredoxins via activation by phosphorylation followed by a thiol-mediated reduction step. It has been speculated that Sulphiredoxin is also involved in the repair of proteins containing cysteine-sulphinic acid modifications [24]. 

In higher organisms, there is evidence for diversity in the response to oxidative stress. Particularly in plants, the production of O_2_ by photosynthesis represents a major potential source of oxidative stress. Furthermore, the generation of H_2_O_2_ in response to various pathogens can elicit localized cell death to limit pathogen spread [25] and thereby serves as part of a systemic response involving the induction of defense genes regulating plant immunity [26]. Therefore, in plants, regulatory systems are required to minimize ROS production without decreasing photosynthetic activity or inhibiting the light-driven production of ROS that is an important signaling molecule controlling plant growth and development [27]. The most abundant ROS scavengers in plants, ascorbate (AsA) and GSH, are typically concentrated in chloroplasts. These metabolites function together in the AsA-GSH cycle to metabolize H_2_O_2_ and thereby to dissipate excess excitation energy in chloroplasts. Moreover, the AsA-GSH detoxification pathway cross-talks with other detoxification pathways including the peroxiredoxin (PRX) and glutathione peroxidase (GPX) pathways which are also important for the detoxification of lipid peroxides. It has been speculated that all these detoxification pathways are tailored to suit specific stressors and that their relative importance probably varies according to the prevailing environmental conditions [27]. 

In animals, there is evidence for species-specific differences in the exploitation of the effects of ROS. For example, in echinoderms fertilization triggers a burst of extracellular production of H_2_O_2_ by a plasma membrane NADPH oxidase with a simultaneous release of ovoperoxidase. This elevated H_2_O_2_ is the oxidant responsible for the extracellular cross-linking reaction involving in the formation of a protective envelope around the freshly fertilized oocyte [28]. 

In insects as in plants, reactive oxygen species can also function as immune effector molecules which exert microbicidal activity. In *Drosophila*, a burst of ROS is generated by DUOX (dual oxidase) upon gut microbe infection and regulates the production of antimicrobial peptides (AMPs) by the fat body, a major immune organ in the fly [29]. As in mammals, the ROS dependent mechanism which activates antimicrobial peptide production involves the activation of the Toll and the NF-κB pathways which both play essential roles in antibacterial and antifungal responses [29,30]. At the same time, the pathogen-induced ROS levels activate the JAK-STAT (Janus kinase–signal transducers and activators of transcription) and JNK (c-Jun NH_2_ terminal kinase) pathways to induce stem cell proliferation counteracting the cellular damage generated by the burst of ROS [31]. 

Another mechanism underlying oxidative stress tolerance in *Drosophila* that influences life-span and xenobiotic response is the conserved Keap1 (Kelch-like ECH-associated protein (1)/Nrf2 (NF-E2-related factor (2) signaling pathway [32]. The Keap1/Nrf2 dimer activated by ROS plays a crucial role in reducing oxidative stress in the germline stem cells from *Drosophila* testis. This pathway regulates the expression of antioxidant and detoxification genes [32,33] and has also been shown to play a critical role in ROS detoxification in mammalian systems.

### 1.1. ROS Regulation of Gene Expression in Vertebrate Systems

Many studies have focused on the regulation of gene expression by ROS in mammalian systems. It is well known that a moderate level of ROS synthesis is physiologically normal and acts as a specific signal in the control of cell proliferation, blood circulation, myoblast differentiation and the regulation of immune and endocrine processes [34]. However, external factors such as xenobiotics and UV-radiation as well as hormones, cytokines, and other physiological stimuli can enhance the production of cellular ROS up to toxic levels [34,35,36]. To protect against the potentially damaging effects of ROS, mammalian cells can enhance the level of endogenous non-enzymatic antioxidant metabolites such as lipoic acid, glutathione, L-arginine and coenzyme Q10 [37] as well as inducing the synthesis of several antioxidant enzymes including superoxide dismutase, catalase and glutathione peroxidase. The mechanism underlying this transcriptional response to ROS involves the activation of MAP protein kinases and redox-regulated transcription factors such as c-Jun, ATF2, ATF4, NFκB, Nrf2 and p53.

Mitogen-activated protein kinases (MAPKs) have been reported to orchestrate ROS-responsive signaling pathways by activating redox-responsive transcription factors such as AP-1, NFκB/IκB and the Nrf2/Keap1 systems. The prevention of ROS accumulation by antioxidants blocks MAPK activation [38,39]. MAPK activation consists of a kinase cascade initiated by a MAPK kinase kinase (MAPKKK), that phosphorylates and thereby activates a MAPK kinase (MEK, or MKK), which in turn phosphorylates and activates one or more MAPKs. In vertebrates, three subtypes of MAPKs have been described. The extracellular signal-regulated kinases (ERKs), the c-Jun N-terminal kinases (JNKs), and the p38 MAPKs. MAPKs can be activated by a wide variety of different stimuli, but in general, ERK-1 and ERK-2 are preferentially activated in response to growth factors, while the JNKs and p38 MAPKs are more responsive to stress stimuli including elevated levels of ROS. The p38 MAPKs represent stress-activated protein kinases activated by extracellular stress and cytokines, (e.g., tumor necrosis factor-a (TNF-α) and interleukin-1b (IL-1β)), and are thereby involved in the inflammation response [40].

The stress-activated protein kinases JNKs, were originally identified by their ability to activate the transcription factor c-Jun via phosphorylation of its transactivation domain [41]. However, it is now clear that they also phosphorylate other target proteins. A key question concerns precisely how ROS activates the JNK and p38 MAPK pathways. Redox-sensitive proteins, such as Trx and glutaredoxin (Grx) have been implicated in this regulatory mechanism [42]. For example, the oxidation of Trx by ROS results in dissociation of the Trx/ASK-1 complex leading to the activation of the two stress-responsive MAPK pathways [42]. ASK1 has been extensively characterized as a ROS-responsive kinase [43,44]. 

The protooncoprotein c-Jun, as well as ATF2 (Activating transcription factor 2) belong to the activating protein-1 (AP1) transcription factor family. These factors regulate gene expression in the context of homo- or hetero-dimeric complexes with other AP1 members (e.g., CREB, Fos, Maf, Jun-B and Jun-D) [45,46,47,48]. AP1 dimers containing ATF2 and c-Jun have been reported to bind to the promoter consensus sequence T^G^/_T_ACNTCA that is encountered in the promoters of many genes involved in DNA repair and apoptosis [49]. 

ATF2 was originally identified from a human brain cDNA library screen as a CRE-binding protein [50]. It is activated by various stimuli including oxidative stress, growth factors, ultraviolet (UV) radiation, and cytokines. ATF2 can shuttle between the nucleus and cytoplasm under basal conditions and following stress stimuli via an autoinhibition mechanism. Specifically, in the inactive state, the ATF2 N-terminal transcriptional activation domain (TAD) interacts with its C-terminal basic leucine zipper (bZIP) DNA-binding domain, inhibiting the ability of ATF2 to activate transcription. Following stress stimuli, ATF2 undergoes phosphorylation at threonine (T69, T71) mediated by stress-activated protein kinases (e.g., p38 and JNK) and is able to translocate to the nucleus as homo- or hetero-dimers with other AP1 transcription factors to modulate the expression of hundreds of genes [47,51]. The proto-oncogene c-Jun is the cellular homolog of the viral oncoprotein v-jun discovered in the avian sarcoma virus 17 [48,52,53]. The c-Jun protein was originally described as a driver of malignant transformation. One characteristic of c-Jun is that it can activate its own expression [54] and therefore can drive a positive autoregulatory loop. It has been shown that in response to oxidative damage, c-Jun is phosphorylated at N-terminal serine residues (S63, S73) by JNK [41,55,56] and that c-Jun play an important role in cell cycle re-entry after DNA damage induced by UV exposure since immortalized fibroblasts lacking c-Jun undergo a prolonged UV-induced growth arrest [57].

The nuclear factor NF-κB is a widely investigated dimeric transcription factor involved in the regulation of genes that control various aspects of the immune and inflammatory response and is responsive to ROS. Redox signaling plays a critical role in NF-κB activation by various stimuli via thioredoxin peroxidases [23]. In unstimulated cells, NF-κB dimers are sequestered in the cytosol through noncovalent interactions with inhibitory proteins termed IκBs [58]. The nuclear translocation and activation of NF-κB as a transcription factor by cytokines, microbial agents, oxidative challenge (ROS) and irradiation occurs through the signal-induced phosphorylation of IκB and its proteolytic degradation by thioredoxin peroxidases. IκB degradation exposes the nuclear localization signal on NF-κB, which allows its nuclear translocation and activation of the transcription of its target genes. Interestingly, the DNA binding function of NF-κB is also regulated by the intracellular redox status. Specifically, the p50 subunit is targeted by S-glutathionylation which reversibly inhibits its DNA binding activity [59].

One of those NF-κB target genes which includes in its promoter the GGGDNWTTCC enhancer element, is the redox-regulating thioredoxin gene (Trx). Trx is an oxidoreductase that works together with the glutathione system to establish and maintain a reduced intracellular redox state. Other NF-κB target genes that play a protective antioxidant role include the peroxiredoxin, heme oxygenase-1, the cystine transporter xc2 and manganese SOD (mnSOD) genes [60]. 

The redox stress-sensitive transcription factor Nrf2 (nuclear erythroid-derived 2-like) regulates the expression of several antioxidant and detoxification genes. In the absence of ROS, Nrf2 is retained in the cytoplasmic compartment in complex with another protein, KEAP1, ensuring effective Nrf2 repression. Upon oxidative stress increase, NRF2 protein is rapidly released from KEAP1 and thereby translocated into the nucleus of affected cells. Nuclear Nrf2 heterodimerizes with Maf and binds to the antioxidant response element ARE sequence (ARE; 5′-^A^/_G_TGA^C^/_G_NNNGC^A^/_G_-3′) located in the promoter regions of antioxidant and detoxification enzymes and by activating expression of these target genes, counteracts oxidative stress [61,62,63]. Specific Nrf2-regulatory targets include elements of the glutathione and thioredoxin antioxidant systems, as well as enzymes catalyzing the detoxification of exogenous and endogenous products, NADPH regeneration and heme metabolism. Consistently, loss of Nrf2 function is associated with increased susceptibility to many environmental stressors. Recently, it has been demonstrated that Nrf2 can cooperate in a ROS detoxification program with the cAMP responsive transcription factor ATF4 [64]. Moreover, Nrf2 is involved in other cellular processes such as autophagy, metabolism, stem cell quiescence and unfolding protein responses [65]. More recently, De Nicola and colleagues [66] have pointed out a potential link between the “reduced” cellular environment and tumor initiation. They have shown that oncogene-mediated induction of Nrf2 in mice promotes ROS detoxification required for tumor initiation [66]. Furthermore, Nrf2 mutations have been isolated from patients with lung, gall bladder, head and neck cancers supporting a pro-tumorigenic role for Nrf2 [62].

The cytokine responsive transcription factor STAT3 has also been shown to be regulated by the redox sensor peroxiredoxin 2 (Prx2) [67]. Specifically, Prx2 act as a sensitive receptor for H_2_O_2_ and transmits oxidative equivalents to STAT3. This, in turn, induces the formation of STAT3 oligomers with reduced transcriptional activity. This observation is consistent with reports that ROS plays a key role in the regulation of tissue regeneration and development [68].

The tumor-suppressor p53 is potently induced by oxidative stress and mediates all the antiproliferative cellular responses to oxidative signals, including transient cell-cycle arrest, cellular senescence and apoptosis [69,70]. Specifically, after oxidative stress the p53 transcriptional response is dependent on the p66Shc protein, the redox enzyme implicated in ROS generation and the translation of oxidative signals into apoptosis [71,72]. The tumor suppressor p53 is critically involved in oxidative stress-dependent apoptosis and is upregulated upon treatment with H_2_O_2_ and UV [73]. Strikingly, p53−/− MEFs show resistance to UV and H_2_O_2_-induced apoptosis [70]. p53 activation leads to a significant increase in ROS levels and apoptosome assembly via the release of cytochrome C from the mitochondria. This release of cytochrome C upon oxidative stress is p66Shc-dependent since p66Shc−/− cells fail to increase ROS levels. Therefore, p66Shc serves as a downstream effector of p53 [72]. However, p66Shc is not involved in p53 functions such as cell cycle arrest but it does regulate p53-dependent apoptosis pointing to a crucial role for p66Shc and p53 crosstalk in the regulation of intracellular ROS levels [72].

### 1.2. Circadian Clocks and Timing of the Response to ROS 

Close links exist between oxidative stress and the circadian clock. It has been speculated that during the origin of life on earth, one of the first driving forces for the evolution of an internal timing mechanism, was the great oxidation event that occurred following the evolution of green plants and photosynthesis [74]. Hydrogen peroxide production and scavenging are strongly time-of-day dependent, therefore, the evolution of an endogenous 24 h clock mechanism was a fundamental step enabling a temporally coordinated homeostatic response to ROS.

The circadian clock is a highly conserved mechanism, from cyanobacteria to humans, that regulates rhythms with a period of 24 h in almost every aspect of biology and behavior. The circadian clock is regularly “reset” or “entrained” by external time cues including light, food and temperature (so-called zeitgebers, time givers) to remain synchronized with the external environment. However, its timing function also persists in the absence of zeitgebers with a rhythm of circa 24 h and based on this property is termed “circadian” (for Latin: circa-diem, around one day). At the molecular level, the vertebrate circadian timing system can be subdivided into three parts: an input pathway that detects and processes zeitgeber information; a core oscillator that is entrained by the input pathway and generates endogenous and self-sustained rhythms; and an output pathway that relays this integrated timing information to various aspects of behavior and physiology [75]. Genetic screens for circadian clock mutants in several model organisms (*Drosophila*, *Neurospora*, *Arabidopsis* and mouse) led to the identification of many genes involved in this mechanism [76,77,78,79,80,81]. Although, the circadian clock genes identified were not conserved between the different groups of organisms, they all share a functional property, namely that they generate circadian rhythmicity by serving as elements of transcriptional-translational feedback loops (TTFL) [81]. In vertebrates, the BMAL and CLOCK basic helix–loop–helix (bHLH), Per-Arnt-Single minded (PAS) transcription factors serve as positive elements of the core TTFL clock mechanism. CLOCK-BMAL hetero-dimers bind to E-box enhancer elements (5′-CACGTG-3′) located in the promoter regions of the negative elements of the TTFL (the period (Per) and cryptochrome (Cry) genes) as well as in the promoters of other clock-controlled genes [82,83], and thereby activate their transcription. The induced PER and CRY proteins in turn form a hetero-dimeric complex and translocate to the nucleus where they inhibit their own transcription by interfering with CLOCK-BMAL driven transcriptional activation [81]. The stability of this core regulatory TTFL is enhanced by additional feedback loops [84]. For their discovery of the first elements of the molecular mechanisms generating circadian rhythms, Michael Rosbash, Michael W. Young and Jeffrey C. Hall obtained the Nobel prize in Physiology and Medicine in 2017. 

In mammals, cell autonomous clocks located in most cell types and tissues (so-called peripheral clocks) are light-synchronized via systemic signals provided by a specialized clock located in the SCN (Suprachiasmatic nucleus) of the hypothalamus, a paired neuronal structure located in the anteroventral hypothalamus above the optic chiasm that is synchronized by light via light-dependent input from the retina-hypothalamic tract [85,86]. In lower vertebrates, including fish and insects such as *Drosophila*, peripheral clocks are reset directly by light without the presence of a light entrainable central pacemaker [87].

Many lines of evidence point to extensive links between the circadian clock and the redox state of the cell. Thus, model organisms with genetically disrupted circadian clocks show many features of abnormal metabolism, such as obesity and diabetes [88,89,90,91]. Furthermore, the early aging phenotype observed in the clock protein BMAL1 knockout mouse has been attributed to an accumulation of ROS due to mitochondrial uncoupling [92,93]. 

The core clock mechanism has been shown to respond to redox state in a range of model organisms [94,95]. This enables the circadian clock to respond to changes in metabolic activity [96,97]. In vertebrates, a feedback loop links redox homeostasis and clock function. The circadian clock controls the NAD pathway via regulation of the enzyme NAMPT, crucial for the synthesis of NAD [98]. This regulation governs the cellular NAD^+^:NADH ratio [99]. Conversely, a NAD^+^-dependent deacetylase, the protein SIRT1, directly regulates the expression of clock and clock-controlled genes via deacetylation of clock proteins and histones [100,101]. The mechanism of protein deacetylation by SIRT1 is dependent upon the availability of NAD^+^. Both NADH and NADPH enhance the binding of CLOCK:BMAL1 and NPAS2:BMAL1 hetero-dimers to the E-box enhancer element, whereas NAD^+^ and NADP^+^ inhibit this activity [102].

It has been shown that circadian clock regulation of the redox stress-sensitive transcription factor NRF2 occurs via the E-box enhancer element. NRF2 regulates the circadian rhythmic expression of antioxidant genes, and genes involved in NADPH production [103]. Consistent with a clock control of ROS homeostasis, time of day dependent differences in the levels of DNA damage, lipid peroxidation and protein oxidation have been documented [104,105]. 

The clock also regulates the production of the hormone melatonin, a potent antioxidant molecule [106]. Melatonin can act as a direct free radical scavenger or as an indirect antioxidant by stimulating antioxidant enzymes including SOD, GPX, and glutathione reductase [107]. Moreover, melatonin is able to regulate the mitochondrial electron transport chain, thereby reducing electron leakage and free radical generation [107]. In addition, it has also been hypothesized that intracellular melatonin can directly neutralize H_2_O_2_ by generating N’-acetyl-5-methoxikynuramine (AFMK) [107]. 

### 1.3. Stress Response and Cytoplasmic Granules 

Following exposure to environmental stress, cells can undergo two alternative fates, either inducing apoptosis or cell cycle arrest to repair the stress-induced damage. The activation of one or the other mechanism is strictly dependent on the extent of cellular damage. These processes minimize cell loss and prevent the survival of cells with genetic and protein alterations. An additional evolutionary conserved strategy for dealing with an imbalanced redox state is Stress Granule (SG) formation. Stress Granules are cytoplasmic non-membrane bound aggregates of RNA and proteins whose formation is associated with inhibition of translation initiation and the disassembly of polysomes [108].

In mammalian cells, stress granule formation is orchestrated by PI3K and p38MAPK that act in a hierarchical manner to drive mTORC1 activity thus facilitating stress granule assembly [109]. SGs are composed of only 10% of bulk mRNA molecules as well as some non-coding RNA (ncRNAs). Recent transcriptomic analyses reveal that SGs are enriched with long mRNAs that exhibit poor translation efficiency [110]. In addition to RNAs, SGs contain various proteins, including G3BP1, T-cell restricted intracellular antigen-related protein (TIAR), PABP1, RACK1, HDAC6 and Y box binding protein 1 (YB-1). Although the role of each protein in SG assembly is not fully elucidated, each appears to play an essential role in SG-associated functions. For example, YB-1 directly binds to and translationally activates the 5′ untranslated region (UTR) of G3BP1mRNAs, thereby controlling the availability of the G3BP1 nucleator for SG assembly. 

In mammals, irradiation and genotoxic drugs are prone to trigger apoptosis. Instead, arsenite, hydrogen peroxide and heat shock treatment induce stress granule formation, that suppresses ROS elevation and thereby inhibits apoptosis. This antioxidant property results from the function of two key SG components, namely the GTPase-activating protein SH3 domain binding protein 1 (G3BP1) and ubiquitin-specific protease 10 (USP10). Under normal conditions, G3BP1 elevates ROS by inhibiting the antioxidant activity of USP10. However, under oxidative stress or heat shock, G3BP1 and USP10 trigger SG formation, which then activates the antioxidant activity of USP 10 [111]. 

Interestingly, it has been shown that upon oxidative insults, SG assembly is associated with an enhancement of YB-1 protein secretion. Furthermore, an enriched fraction of extracellular YB-1 (exYB-1) caused a G2/M cell cycle arrest in receiving cells thus inhibiting cell proliferation [112]. These observations suggest that the release of extracellular YB-1 can function as a stress-dependent paracrine/autocrine signal that controls cell cycle progression. Despite the considerable literature on SGs in mammals, little is known about how much these membrane-less organelles and their functions are conserved during evolution. Interestingly, it has been shown that the nuclear localization of the SG marker YB-1 is robustly regulated in fish by the circadian clock [113]. Specifically, a daily nuclear entry of YB-1 at the beginning of the light phase appears to be mediated by clock-controlled changes in YB-1 SUMOylation. Moreover, in zebrafish, the YB-1 nuclear protein is able to downregulate cyclin A2 transcript levels thus providing a direct link between the circadian clock, YB-1 and the control of cell proliferation. Thus, in response to oxidative stress, YB-1 seems to play distinct roles depending on its subcellular localization. In the nucleus, YB-1 restrains cell cycle progression while in the cytoplasm it participates in SG assembly and inhibition of translation. Overall, it appears that YB-1 acts to prevent and then eventually repair genotoxic damage. 

Thus, oxidative stress response pathways have been extensively studied in mammalian systems. However, given the diversity of antioxidative stress pathways in other species, are these pathways conserved in all vertebrates or may there be species-specific differences in their function depending on the ecological niche occupied? In the next section, we will explore recent progress made in studying the oxidative stress response in fish.

## 2. Fish as Models for Studying ROS Responses

Recent work has compared the response to oxidative stress in selected species of fish with mammalian models such as human and mouse. Fish represent the most diverse and largest vertebrate group which occupy an important ecological position and have great commercial value. Furthermore, zebrafish and medaka are also powerful genetic models that provide a wealth of molecular and genetic tools for studying various biological processes at the molecular level. Another unique and attractive advantage of using fish for studying ROS pathways, is that their peripheral tissues are directly light responsive [87] and central to these light-driven responses is an increase in intracellular ROS levels [114,115]. Specifically, most tissues and cell types in fish express photoreceptors, directly light-regulated clocks, light enhanced DNA repair capacity and importantly the expression of many genes is induced in fish cells upon direct exposure to visible as well as UV light and ROS. This cell-autonomous property is even observed in cell lines derived from various fish tissues [116,117,118]. Therefore, fish cell lines represent powerful in vitro models for exploring the capacity of light via ROS to regulate physiological mechanisms in vertebrates. The utility of fish also extends to studying the effects of evolution in response to a changing environment. Notable examples are species of blind cavefish that inhabit perpetually dark subterranean environments and exhibit a set of striking anatomical adaptations including eye loss, enhancement of non-visual senses and loss of body pigment, so called troglomorphisms. One particular species, the Somalian cavefish *Phreatichthys andruzzii*, represents one of the most extreme examples of cavefish since it has been completely isolated from surface water and surface-dwelling forms for at least 2–3 million years. Interestingly, this species also exhibits complete loss of light, UV and ROS induced gene expression. Therefore, comparing the responses to ROS in different fish species subjected to different evolutionary pressures, represents a unique approach to gain insight into how the ROS response is shaped by the environment during evolution.

### 2.1. Sunlight, ROS and Regulation of the Circadian Clock in Fish 

A remarkable feature of the organization of the circadian timing system in vertebrates is a fundamental difference in the mechanisms whereby light entrains the multiple peripheral tissue clocks. Thus, while peripheral clocks are directly light-regulated in lower vertebrates such as fish, in modern mammals the photic entrainment of peripheral clocks is dependent upon a centralized, retina-based photoreception system [119]. These observations predict the existence of fundamental changes in the regulatory networks of peripheral clocks over the course of vertebrate evolution. In zebrafish, functional genomic analysis has identified more than 40 opsins of which 32 are non-visual opsins expressed in peripheral organs [85,120]. It has also been shown that exposure to visible light can stimulate H_2_O_2_ production in zebrafish embryonic cell lines and that this production is a key signal for mediating light-dependent circadian gene expression [114,115]. It is also well documented that exposure of cultured mouse, monkey and human cells to violet-blue visible light, as well as UVA, also stimulates H_2_O_2_ production via photoreduction of flavoproteins [121]. Hirayama et al., in 2007 [115], demonstrated that ROS species act as a second messenger coupling photoreception to circadian clock entrainment and consequentially regulate the circadian expression and activity of clock genes as well as antioxidant enzymes such as catalase that is responsible for H_2_O_2_ degradation. A more recent study [114], revealed a key role for NADPH-flavin-containing oxidases (NOXes) in the regulation of light-inducible clock gene expression in zebrafish. Using a pharmacological approach and by studying zebrafish cell lines, visible light was shown to trigger increases in ROS levels via NADPH oxidase activity, which in turn activates the expression of the light regulated clock genes *zfcry1a* and *zfper2*. This induction of *zfcry1a* and *zfper2* gene expression is also dependent on the activation of the JNK and p38MAPK stress pathways. Surprisingly, the promoter enhancer elements targeted by these ROS pathways was not one of the classical enhancers regulated by ROS. Instead, exposure to ROS species, as well as visible and UV light, activated the expression of circadian clock and DNA repair genes via D-box enhancer elements located in their promoters [114,122]. Interestingly, in mammalian cells, neither blue light, UV nor H_2_O_2_ exposure activates gene expression via D-box enhancer elements [114,122]. 

### 2.2. The D-Box and the Transcriptional Response to ROS

The D-box element was first described in mouse liver as one of the transcriptional enhancers present in the albumin gene promoter [123]. In 1990, Uli Schibler’s group cloned the first transcription factor that binds with high specificity to the D-box element of the rat liver albumin promoter, thereby called DBP (D-binding protein) [124]. Subsequently, two other transcription factors were shown to bind to the D-box element and to serve as transcriptional activators, namely the thyrotrophic embryonic factor (TEF) [125] and the hepatocyte leukemia factor (HLF) [126]. DBP, TEF and HLF belong to the so-called PAR bZip (proline and acidic amino acid-rich basic leucine zipper) transcription factor family which share a proline-and acidic amino acid-rich domain positioned proximal to a C-terminal bZip domain as well as an extended basic region at their N-termini and which are highly conserved throughout evolution [127,128]. These transcription factors constitute a subfamily of the basic leucine zipper proteins (bZip), characterized by a C-terminal α-helical region that allows homodimerization or heterodimerization between other bZip proteins [129]. All PAR bZip family members activate transcription of downstream genes by binding as homo- or hetero-dimers to D-box elements matching the consensus sequences RTTAYGTAAY [130]. These transcriptional activators compete for DNA binding with another bZip protein which shares a similar DNA-binding profile but which lacks the PAR domain and functions as a repressor, namely E4BP4 (E 4 binding protein 4) [129,131]. 

**Figure 1:**
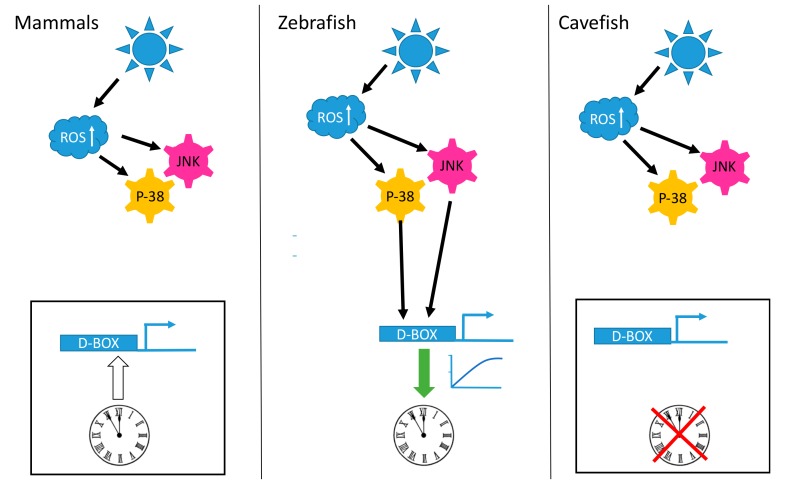
Light, reactive oxygen species (ROS) and the D-box enhancer. Schematic representation of how D-box enhancer-driven gene expression is differentially influenced by blue light exposure in mammalian, zebrafish and cavefish cells. In all three cell types light triggers an increase in intracellular ROS levels that in turn activates the p-38 mitogen-activated protein kinase (MAPK) and c-Jun NH_2_ terminal kinase (JNK) stress pathways. In zebrafish cells (central panel), this signaling results in the activation of D-box-driven gene expression, ultimately leading to circadian clock entrainment (indicated by the green arrow). In mammalian cells (left panel) and in cavefish cells (right panel) this signaling fails to activate gene expression via the D-box enhancer element and does not entrain the circadian clock. The white arrow starting from the clock and pointing on the D-box element indicates the circadian clock regulation of this enhancer in mammals [132]. The red cross over the clock in the cavefish cells indicates the blind circadian clock observed in these cells [141].

A striking observation is that the D-box enhancer element operates in a completely different fashion in mammalian cells compared with fish cells. In mammalian species, the D-box is not involved in the light input pathway of the clock. The expression of genes which contain D-boxes in their promoters do not respond to visible light, UV or ROS exposure (Figure 1). Instead, the mammalian D-box is clock regulated and thereby plays a role in the activation of genes belonging to the output pathway of the clock machinery [132]. The expression of both DBP [133] and HLF [134] is strongly clock regulated in the mammalian liver and the D-box enhancer has been shown to mediate the clock-driven regulation of genes encoding enzymes such as CAT and SOD [115,135] as well as the production of low molecular weight antioxidants such as glutathione (GSH) [136,137,138] with important functions in the defense against xenobiotic and oxidative stress [139]. 

In contrast, D-box regulation in fish is tightly linked with coordinating the gene expression response to oxidative stress as well as sunlight. Furthermore, as a result of genome duplication events in early teleost ancestors, fish species possess extra D-box binding factors (TEF1, TEF2, HLF1, HLF2, DBP1, DBP2 and 6 E4BP4 homologs) [140]. These factors exhibit significant differences in their tissue- and cell type-specific expression patterns and also can bind to D-boxes as hetero- or homo-dimers. Therefore, it is clear that in fish, transcriptional regulation at the D-box is inherently complex. A clear challenge for the future is to identify precisely how elevated ROS levels lead to the regulation of this complex combination of transcription factors via activated JNK and p38 MAPK activity.

### 2.3. Adaptation of Mechanisms Responding to Oxidative Stress during Evolution

**Figure 2:**
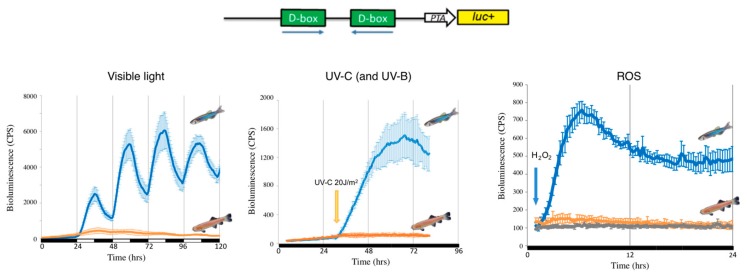
Loss of D-box function in cavefish. Representative in vivo bioluminescence assays performed in zebrafish (blue traces) and cavefish (orange traces) of cells transfected with the D-box enhancer luciferase reporter derived from the zebrafish *6-4* *phr* promoter [122] (upper part of the figure). Cells were exposed to three different stressors: light-dark cycles (left panel), a UVC pulse (central panel) or 300 μM H_2_O_2_ (right panel). The grey (right panel) trace indicates luciferase expression of cavefish cells not exposed to H_2_O_2_ (negative controls). Black and white bars below each panel indicate the lighting conditions.

Insight into how the light-ROS-D-box signaling pathway has adapted during evolution under extreme environmental conditions has been gained from a set of comparative studies focusing on a species of blind cavefish (*Phreatichthys andruzzii*). This species has evolved over 2–3 million years completely isolated from sunlight, in layers of water locked beneath the Somalian desert [114,122]. Like other species inhabiting perpetually dark cave environments, these cavefish exhibit a set of striking anatomical adaptations including complete eye loss and absence of body pigmentation, so-called troglomorphisms. By comparing cavefish- and zebrafish-derived cell lines, it was revealed that light entrainment of the clock as well as photoreactivation DNA repair has also been lost during evolution of this cavefish [122,141,142]. Specifically, this originates from a loss of light-, UV- and ROS-induced gene expression. Similar to the situation in zebrafish, blue or UV light triggers an increase in cellular ROS levels as well as an activation of the MAP kinase stress pathways in the cavefish cell lines. However, these events do not result in the transcriptional activation of D-box enhancer-regulated clock genes such as *per2* and *cry1a* or the *CPD*, *DASH* and *6-4 photolyase* DNA-repair genes (Figure 1 and Figure 2). Precisely how the D-box regulatory factors have been modified during cavefish evolution remains unclear. Furthermore, it will be fascinating to compare these cavefish regulatory mechanisms with those of mammals which also fail to respond to ROS.

Interestingly, the cavefish *P. andruzzii* appears to be only species described to date, apart from placental mammals, that lacks the highly evolutionary conserved photoreactivation DNA repair function. It has been speculated that in the DNA repair systems of *P. andruzzii*, we are witnessing the first stages of a process that occurred previously in the ancestors of placental mammals during the Mesozoic era. This speculation is based on the ‘‘nocturnal bottleneck’’ theory [143,144,145]. This theory predicts that the ancestors of modern mammals became exclusively nocturnal in order to avoid predation by diurnal carnivorous dinosaurs. This adaptation to a dark ecological niche may also explain many features of present-day mammals including a general loss of extraretinal photoreception, as well as adaptations in the eye and retina to facilitate vision under low-lighting conditions [143,145]. Adaptation to a nocturnal lifestyle is also predicted to have entailed a general loss of light-dependent repair mechanisms that target UV-induced DNA damage, namely the photolyase genes and photoreactivation function [143]. It is also interesting to note that similar to cavefish cells, in mammalian cell lines light and UV exposure all trigger an increase in cellular ROS levels followed by activation of the MAP kinase stress pathways but this does not result in activation of D-box enhancer-mediated gene expression [114,122]. 

Another example where changes in the response to ROS are coupled with evolution in an extreme environment is the naked blind mole rat (*Spalax*). These are small subterranean rodents common in the Middle East that are distinguished by their adaptation to life in a hypoxic underground environment. Specifically, these animals undergo cycles of digging closer to the surface and then burrowing in their subterranean tunnel systems. As a consequence, at the cellular level, these animals experience cycles of hypoxia followed by reoxygenation. In a normal animal, these alternating shifts in oxygen levels can serve as a source of genomic instability, which underlies both aging and cancer. Severe hypoxia results in S-phase arrest, dNTPs depletion and replication stress as well as a general repression of DNA repair activity. Then, upon reoxygenation, S-phase restarts during an initial period when DNA repair has still not recovered to normal levels. Therefore, DNA replication occurs in the presence of ROS-induced DNA damage, leading to the accumulation of mutations. [146,147]. However, *Spalax* exhibits remarkable longevity and a striking resistance to cancer [148]. It is therefore evident that a key aspect of the adaptations *Spalax* has made for life in its hypoxic environment are fundamental changes in the molecular mechanisms responding to cycles of hypoxia and oxidative stress. It has been speculated that the cancer resistance in *Spalax* results from an amino acid substitution in the p53 gene leading to a R174K substitution. p53 serves as a master regulator of the DNA damage response and has been termed the “guardian of the genome.” This amino acid substitution in *Spalax* impairs the ability of the p53 protein to trigger apoptosis, although the protein is still able to induce cell cycle arrest [149].

## 3. Conclusions

Our current understanding of the molecular mechanisms responding to oxidative stress reveals extensive conservation of many of the main regulatory pathways between organisms as diverse as mammals, yeast and *Drosophila*. However, there are also examples of fundamental changes in these mechanisms during evolution. For example, the switch in the function of the D-box enhancer from a ROS regulatory target in fish, to a clock regulatory target in mammals as well as the loss of ROS responsiveness of the D-box enhancer during cavefish evolution. Furthermore, fundamental changes in the functionality of the p53 protein have accompanied the adaptation of the naked blind mole rat to its life in a hypoxic environment. These findings reveal that the functions of ROS responsive mechanisms are also potentially plastic and are shaped by the selective pressure of the environment. By identifying precisely which elements of these regulatory mechanisms are “targeted” during the process of evolution, we will gain considerable insight into how organisms adapt to oxidative stress and damage over evolutionary time scales.
